# Neuroarchitecture: How the Perception of Our Surroundings Impacts the Brain

**DOI:** 10.3390/biology13040220

**Published:** 2024-03-28

**Authors:** Sarah Abbas, Nathalie Okdeh, Rabih Roufayel, Hervé Kovacic, Jean-Marc Sabatier, Ziad Fajloun, Ziad Abi Khattar

**Affiliations:** 1 Faculty of Medicine and Medical Sciences, University of Balamand, Kalhat, Tripoli P.O. Box 100, Lebanon; sarah.m.abbass@gmail.com; 2 Faculty of Architecture and Design, Azm University, Azm Educational Campus, Tripoli 1300, Lebanon; 3 Department of Biology, Faculty of Sciences 3, Lebanese University, Campus Michel Slayman Ras Maska, Tripoli 1352, Lebanon; nathalie_okdeh@hotmail.com; 4 College of Engineering and Technology, American University of the Middle East, Egaila 54200, Kuwait; rabih.roufayel@aum.edu.kw; 5 CNRS, INP, Institut Neurophysiopathol, Aix-Marseille Université, 13385 Marseille, France; herve.kovacic@univ-amu.fr (H.K.); sabatier.jm1@gmail.com (J.-M.S.); 6 Laboratory of Applied Biotechnology (LBA3B), Azm Center for Research in Biotechnology and Its Applications, EDST, Lebanese University, Tripoli 1300, Lebanon

**Keywords:** neuroarchitecture, architecture, parahippocampal place area, mirror neurons, place neurons, wayfinding

## Abstract

**Simple Summary:**

This literature review delves into the interdisciplinary field of neuroarchitecture, exploring the significant impact of architectural design on human behavior, emotions, and cognitive processes. It examines the roles of specific brain regions, such as the Anterior Cingulate Cortex (ACC) and the Parahippocampal Place Area (PPA), in perceiving and responding to architectural environments. The review also discusses the influence of mirror neurons in empathetic reactions to architecture, the emotional effects of design elements like natural light and color, and the importance of architectural features in spatial navigation and wayfinding. The paper aims to highlight the profound connection between architectural spaces and neurological functioning, emphasizing architecture’s role in enhancing human well-being.

**Abstract:**

The study of neuroarchitecture is concerned with the significant effects of architecture on human behavior, emotions and thought processes. This review explores the intricate relationship between the brain and perceived environments, focusing on the roles of the anterior cingulate cortex (ACC) and parahippocampal place area (PPA) in processing architectural stimuli. It highlights the importance of mirror neurons in generating empathetic responses to our surroundings and discusses how architectural elements like lighting, color, and space layout significantly impact emotional and cognitive experiences. The review also presents insights into the concept of cognitive maps and spatial navigation, emphasizing the role of architecture in facilitating wayfinding and orientation. Additionally, it addresses how neuroarchitecture can be applied to enhance learning and healing environments, drawing upon principles from the Reggio Emilia approach and considerations for designing spaces for the elderly and those with cognitive impairments. Overall, this review offers a neuroscientific basis for understanding how human cognition, emotions, spatial navigation, and well-being are influenced by architectural design.

## 1. Introduction 

Neuroarchitecture is an interdisciplinary research field that integrates neuroscience and architecture and focuses on how individuals interact with the built environments. Since 2000, the discipline of neuroarchitecture has grown to explain the relationship between the brain and perceived surroundings [1]. Human minds can include aspects of physical and cultural environments, which means that the kind of environments that individuals create can alter their minds and capacity for thought, emotion, and behavior. That being said, how much does the built environment affect individuals’ behavior and well-being? In particular, how does the body and the brain respond to the built environment? Biological sciences and neuroscience are opening promising doors to the essence of the brain, mental functions, and consciousness and can valorize the interaction between architecture and the human mind. Sarah Robinson states that the world is experiencing a neuroscience revolution that stands parallel in significance to Galileo’s impact on physics and Darwin’s on biology [2]. 

While many people often assess buildings mainly in terms of their functional utility, physical comfort, economic value, symbolic importance, or aesthetic appeal, architecture actually serves a far deeper role in human life [2]. Finnish architect Juhani Pallasmaa argues that the significance of buildings goes beyond these material and superficial aspects. Rather than just offering physical protection and facilitating various activities, buildings also play a vital role in housing our mental lives—our thoughts, memories, desires, and dreams. Pallasmaa describes buildings as important extensions of ourselves, both individually and collectively. They act as mediators between our consciousness and the world around us, helping to “internalize the world and externalize the mind”. According to his view, architecture is not just a physical structure; it is also a manifestation of our mental space, which in turn is influenced by the architecture around us. 

This reciprocal relationship is captured in Maurice Merleau-Ponty’s phenomenological concept of “the chiasmatic bind”. This idea highlights the interconnectedness between our physical and mental spaces and the world, suggesting a dialectical relationship where each influence and is influenced by the other [3]. 

Evan Thompson describes our human existence as one of “radical embodiment” where the “subject of experience and feeling is not just the brain or even the brain combined with the body, but a human being situated within a social and cultural context” [4]. He goes on to argue that culture is not an external force modifying our biological blueprint; rather, it is integrated into the fabric of our minds from the very beginning. 

Therefore, this paper aims to investigate the impact of architectural spaces on individuals. It hypothesizes that architectural design significantly influences human neurological and psychological processes, positing that specific elements of the built environment, such as spatial layout, natural light, and color schemes, directly impact cognitive functioning, emotional well-being, and spatial navigation. To investigate this hypothesis, this literature review was structured into three main sections: the first section explores the neural mechanisms involved in perceiving spaces, the second section explores the neural systems helping us navigate built environments, and the last section investigates how surrounding spaces influence individuals’ behavior, emotions, and mental well-being. By examining the roles of key brain areas, including the anterior cingulate cortex (ACC) and the parahippocampal place area (PPA), and the concept of mirror neurons, this literature review aims to demonstrate how architecture can transcend its traditional utility to become an integral component of mental and emotional health.

## 2. Architecture Perception: Role of Parahippocampal Place Area (PPA) and Anterior Cingulate Cortex (ACC)

Understanding how spaces impact individuals’ brain structure and behavior has been a core research point for architects and environmental psychologists [5]. Architects consider a design’s impact on residents while mapping out the structure (e.g., adequate lighting) [1]. They can strategically design spaces to enhance creativity, cognition, concentration [6], and memory [7]. As Fred Gage stated, “Changes in the environment change the brain, and therefore they change our behavior. In planning the environments in which we live, architectural design changes our brain and our behavior” [5]. In accordance with this, numerous studies have explored the structural and functional changes in the brain in relation to how the surrounding environment is perceived. 

### 2.1. How the Brain Perceives Our Surroundings: From Ambiguity to a Clear Image 

**Figure 1:**
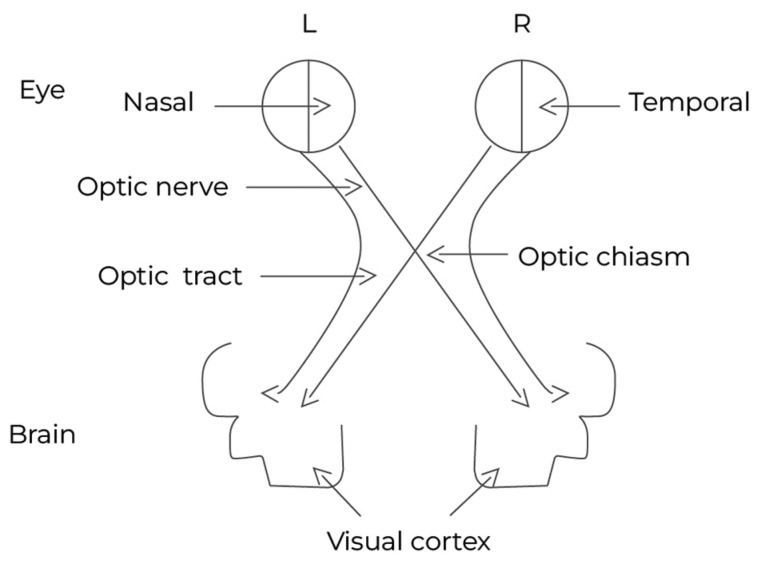
Human visual pathway. The light hits the retina in the left (L) eye and in the right (R) eye, creating a message that crosses over into the optic chiasm via the optic nerves to reach the visual cortex.

The brain adapts to the visual perception of surroundings through a variety of processes [8]. Whenever the individual is presented with a visual image, the human brain reacts with a wave of activity [8]. This reaction begins when the light hits the eye’s retina, which creates a message. The nerve fibers of each eye transport the latter, which cross over at the optic chiasm and into the brain via the optic nerve [9], as seen in Figure 1. 

The brain then processes this image but sometimes establishes sensations that over-ride the initial visual input if deemed improbable [10]. Consequently, the human visual system can often successfully construct a stable representation of the image even when it receives incomplete or ambiguous information [when our brain is impressed but we do not notice it]. Perception is, therefore, inherently a process of resolving ambiguities. The perceptual system typically integrates local clues to arrive at the most likely global interpretation of the retinal input [11].

The visual system is suggested to be arranged hierarchically, with the posterior visual cortex responding earlier than higher anterior visual cortical areas [12]. According to this theory, the early visual cortex responds primarily to big and complex visual patterns, such as faces and houses, while the high-level visual cortex is driven by more localized visual elements, such as contours, edges, and limits [8]. Besides the objects’ shapes, the light levels were found to affect the perception and understanding of images, which is evident in the perception of a black-and-white image. The more colored an image is, the more likely our brains will perceive it better and understand its context [13].

**Figure 2:**
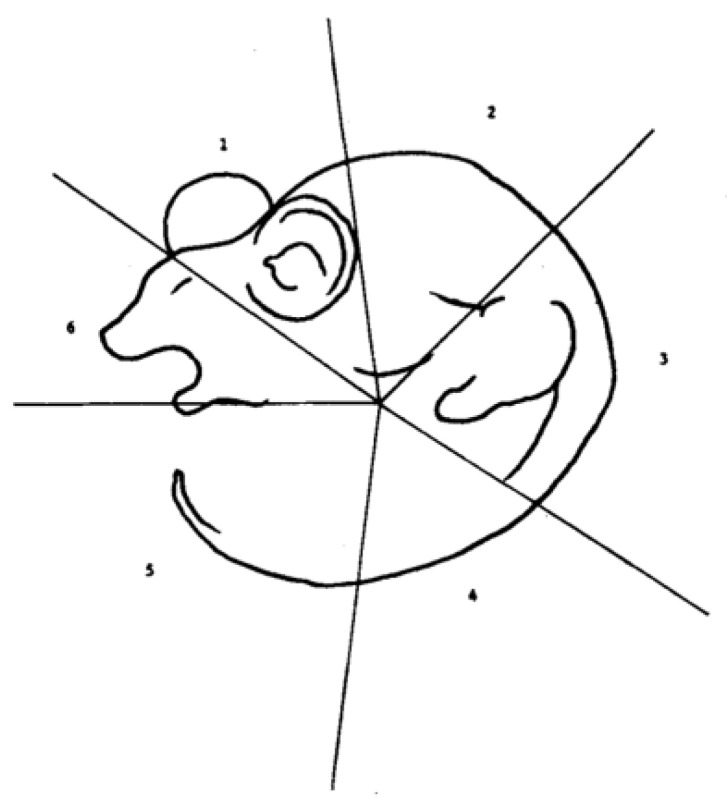
The rat–man figure with segments shown (1, 2, 3, 4, 5, and 6) per the study conducted by Chastain and Burnham [14].

However, if the perception of our surroundings were to be categorized into stages, the computation of lightness would be involved in the secondary processing stages. In contrast, the first stages focus on the extraction of the contours of objects, and the last stages are related to processing the objects’ colors [11]. Accordingly, what the brain sees first from an image significantly impacts how the brain interprets the entirety of an image. To test this hypothesis, a study was conducted on 36 subjects with an ambiguous rat–man image (Figure 2) in successive segments. Starting segments were divided into three: one which would produce the perception of a rat, the other of a man, and the third of either a man or a rat. The first segment was shown to have a statistically significant impact on image perception; however, this significance was reinforced by following the first segment with some additional segments to reach the desired outcome [14].

Therefore, this research showed that the perceiver analyzes the segment shown initially and formulates a hypothesis. The individual then tries to apply this first theory to interpreting the other segments. The first single segment generates various initial hypotheses, and additional segments must come after the various starting segments for the constructive processes to fully become perceptually distinct images. Thus, the opening sections lay the groundwork for a theory, strategy, model, or framework that will direct the rest of the image perception processes [14].

From ambiguous figures to thoroughly dissected images, the human brain holds immense power in perceiving and understanding its surroundings. Thus, architectural designs are primarily directed to individuals’ sense of vision. The Canadian designer Bruce Mau says this: “We have allowed two of our sensory domains—sight and sound—to dominate our design imagination. In fact, when it comes to the culture of architecture and design, we create and produce almost exclusively for one sense—the visual” [15]. Therefore, it is essential to understand which areas of the brain are involved in these processes.

### 2.2. Role of Parahippocampal Place Area (PPA) and Anterior Cingulate Cortex (ACC)

**Figure 3:**
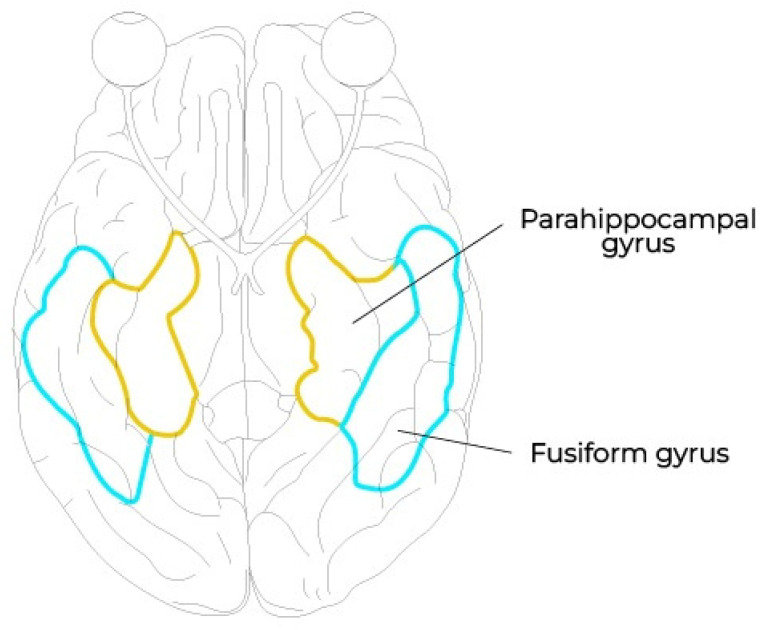
Parahippocampal gyrus (yellow) and fusiform gyrus (blue).

The hippocampus has been shown to have a key role in perception, as its removal caused rats to lose their reference spatial map, which led to behavioral deficits [16]. The parahippocampal cortex (PHC) (shown in Figure 3) is thought to be crucial for both memory and visuospatial cognition [17]. More specifically, the parahippocampal place area, located in the posterior part of the PHC, seems to be the most involved in the perception of architectural spaces [18].

A study conducted by Nancy Kanwisher and her colleagues laid the foundation for a connection between the brain and encounters with architecture. It attributed this connection to the parahippocampal place area (PPA) [18]. They observed that PPA activity (1) is unaffected by the subjects’ familiarity with the location shown, (2) does not rise when subjects perceive motion within the scene, and (3) is higher while viewing new versus repeated sights. The PPA was substantially more active when respondents looked at complex images, such as rooms with furniture, scenery, and city streets, than when they looked at photographs of items, faces, houses, or other visual stimuli [18]. 

Accordingly, in another study, fMRI data were used to understand how the human brain codes the perception of landmarks. Individuals were studied while viewing the interior and exterior of campus buildings. The results showed that the parahippocampal place area (PPA), retrosplenial complex (RSC), and occipital place area (OPA) were activated similarly in response to interiors and exteriors even though they are visually different [19]. Moreover, a different response within the PPA was noted in individuals seeing different landmarks, underscoring the importance of real-world experience with the landmark.

Regardless, visual experience alone, outside of real-world experience, was still enough to activate the PPA [19], which has been linked to landmark recognition in the past because of its strong responses to sceneries and buildings [20,21] and items that fit into the criteria for landmarks [22].

The role of the PPA in perceiving architectural spaces was further studied in two human fMRI experiments, where it was demonstrated that the PPA and the OPA processed the borders of scenes based on the observer’s space rather than the physical environment. The PPA activation was also shown to be more sensitive to changes related to mid-level perceptual characteristics of scenes (such as the rectilinear pattern of window frames) [23]. Furthermore, the PPA seems to be view-point specific [24] and primarily focuses on the perception of an immediate scene [25].

Another study by Banaei et al. investigated the cognitive processes and brain dynamics of individuals walking through different architectural spaces using virtual reality (VR). The study found the anterior cingulate cortex (ACC) plays a vital role in the perception of architectural spaces. The ACC was activated when individuals walked through a built environment and was significantly activated when individuals faced curvature forms. Moreover, the posterior cingulate cortex and the occipital lobe were shown to be involved in the perception of different perspectives and changes in room depths. However, small changes in perception between different walls of the same architectural space did not appear to cause any significant difference within the brain [26].

Therefore, there are neural mechanisms set in place to help us make sense of our surroundings and our perception even if what we are perceiving is not clear [14]. Additionally, it was shown that the parahippocampal place area (PPA) and the anterior cingulate cortex (ACC) are majorly involved in the mechanism of perception [24,26]. However, this only explains how we understand what we are seeing; how do we know how to navigate the perceived spaces?

## 3. Spatial Navigation, Wayfinding, and the Hippocampus

In the process of spatial navigation, humans and animals rely on landmarks, including architectural elements, to determine their location in the world and navigate towards their destination. This wayfinding strategy is known as landmark-based piloting [27]. In large-scale environments, navigation requires decision-making regarding the direction to take. Opting for an unfamiliar route may occur when, for instance, there is no prior experience traveling between two locations or when the regular path is obstructed. This suggests the existence of a comprehensive representation, often termed a “cognitive map”, where landmark positions are depicted in relation to their spatial connections to each other [28]. The hippocampal system is crucial for the retrieval of declarative memories that include information about both locations and the events occurring in those locations [29]. The hippocampus, therefore, plays a major role in navigation and encoding these “cognitive maps” [30].

### 3.1. Place Neurons: Spatial Navigation in the Built Environment

Episodic memory is the ability to acquire, store, and recall personal events or experiences that are situated in a particular time and place [31]. Studies have shown that the loss of episodic memory, or amnesia, is due to hippocampal damage [32]. This relates to the claim that the hippocampus is responsible for encoding a “cognitive map”. This kind of map can be utilized to detail the formation of discrete episodes from the diverse elements of a memory, or how spatial or architectural landmarks construct a map of the environment [33]. This interest in the study of the hippocampal role in episodic memory led to the findings of “place neurons”.

Place neurons, also known as place cells, are neurons found in the hippocampus that are responsible for the brain’s representation of an individual’s location in a particular environment. The discovery of place neurons took place in an experiment led by John O’Keefe and his colleague, where they experimented by placing electrodes in the hippocampi of rats. The experiment concluded that certain cells did not only respond to visual stimuli but were instead activated based on the rats’ specific location within the laboratory [16].

A study showed that place cells exhibited different behaviors when rats foraged for randomly dispersed food in different locations. Place neurons were differently active in different contexts, such as including the visual environment, the configuration of the traversable space, and motor behavior [34]. This conclusion suggests that spatial context and layout have a great impact on the behavior of place cells. The study also suggests that place neurons’ behavior in rationally symmetric environments exhibits firing preferences towards specific walls, likely guided by head direction cells, highlighting the fundamental role of the direction system in mental map formation [35]. In another study, researchers manipulated the walls of a box, observing that distinct cells responded to various wall arrangements. The resulting pattern indicated the cells’ sensitivity to the rat’s proximity to the walls, suggesting a potential distance-measuring mechanism, which is currently believed to involve grid cells [36].

Therefore, the hippocampus is intriguing due to its dual role in temporal navigation through episodic memory in humans and spatial navigation via place cells at the circuit level. Thus, when considering wayfinding in architecture, architects can evaluate how individuals will perceive their journey through a building by analyzing spatial cues provided by its structure [30].

### 3.2. Wayfinding as a Design Tool

The built environment should then support perceptual organization and foster the creation of cognitive schema or neuronal maps. A good example illustrating the importance of neuronal maps can be found in research focused on wayfinding behavior. Wayfinding encompasses sensory perception and spatial cognition, involving activities like positioning oneself in space, choosing a route, selecting pathways from a starting point to a destination, overseeing progress during movement, and identifying the destination upon arrival [37]. Relating to the insights given by the hippocampus and its role in encoding space and the observer’s position through neural maps, it is evident that landmarks and spatial cues in the built environment can be a design approach to improve navigability within a space. These insights bring potential solutions for individuals grappling with memory disorders associated with dementia [6].

In the context of dementia of the Alzheimer’s type (DAT), individuals face challenges related to impaired brain functions, affecting visual motion processing, cognitive mapping, and route-planning abilities. Wayfinding difficulties can be seen as deficits in spatial orientation, including difficulties in self-localization and understanding route instructions. Architectural intervention in this case can be implemented to optimize spaces for individuals with dementia such as the use of wayfinding cues like landmarks. Landmark design considerations include achieving unique colors and relevant placements and adopting dementia-sensitive lighting levels, the meaningful personalization of space, and minimizing decision points and small-scale units that may help reduce spatial disorientation [38].

Having understood the neurological mechanisms involved in perceiving and navigating architectural spaces, this gives us a solid basis to go a step further and explore if and how perceiving these spaces affects our behavior and well-being.

## 4. The Built Environment and Embodied Simulation

The impact of architectural spaces on one’s brain can be understood through the neurological basis of perception. Observing the world is dependent on motor-, somatosensory-, and emotion-related components that all come together to showcase the pragmatic nature of one’s connection with the world. Notably, cortical motor areas are not only motor-centric; they possess sensory properties, responding not only to micro-stimulation-inducing movement but also to visual, tactile, and auditory stimuli. These neurons display embodied motor simulation, indicated when visual, tactile, or auditory stimuli are presented within the peri-personal space associated with the body part controlled by the neurons [39]. Premotor area F4 includes neurons that are responsible for controlling arm reaching and orienting movements of the hand. Interestingly, an experiment by a group at Yale University demonstrated that the same neurons that fire when an arm reaches for something also fire when the arm is being touched, or if an object moves towards the arm [40]. This study thus demonstrated that witnessing an object or event within the peri-personal space triggers the motor simulation to that specific spatial location. Premotor area F5 includes canonical neurons that showcase the same behavior. Interestingly, when a monkey is explicitly directed not to engage in movement but to solely observe the object, the mere sight of the object activates the exact neuron responsible for controlling the grasping action. It is in this area F5 where mirror neurons were first discovered.

### 4.1. Mirror Neurons: Feeling Empathetic toward Buildings and Others

The discovery of mirror neurons took place at the University of Parma in experiments conducted on the neural activities of monkeys. Scientists inserted electrodes in the monkeys’ brains to record the neural activity involved with grasping. Interestingly, they found that certain neurons were activated not only in monkeys that were performing the grasping action but also in those simply watching another monkey carry out the same action. From a motor standpoint, these neurons are the same as canonical neurons. However, what triggers the firing of these neurons perceptually is not the observation of an object; rather, it is the observation of a specific action [41]. This phenomenon indicated that the observing monkeys were neurologically “mimicking” the actions they saw or heard. Hundreds of neuroimaging studies on humans have provided solid evidence that similar neural activities occur in the human premotor cortex and posterior parietal lobe.

A neurophysiological study has also demonstrated the spatial relevance of the mirror mechanisms. An experiment documented the activity of mirror neurons in the hand field of a macaque’s ventral premotor cortex as the monkey observed a visuomotor task performed by an experimenter. Notably, these observations were conducted in both the monkey’s peripersonal and extrapersonal space. The study showed that half of the canonical mirror neurons fired when the action was perceived near and half of them fired when the action was farther away. However, more than half of the recorded canonical and canonical mirror neurons exhibited weak responses to action presentation when it occurred behind a transparent plastic barrier. Nearly half of these neurons showed no significant activation under this condition. This showcased that the spatially constrained encoding of objects, performed by both canonical and canonical-mirror neurons, is dependent on a pragmatic rather than metric representation of space. This significant finding highlights that neuronal responses to objects rely on the actual potential for the monkey to interact with the observed stimulus [42]. This study demonstrates that the frontal and parietal motor areas in the human brain are modulated by proxemics and that the physical locations and environments in which actions are being performed dictate the behavior of these neurons.

Moreover, mirror systems offer insights into our capacity for empathy. For example, they illuminate why one might instinctively touch a part of our body that corresponds to someone else’s injury or why one experiences joy when watching a dancer perform. Essentially, these mirror systems in our brain appear to derive pleasure from neurologically replicating the actions observed in others. This has already been evident in the discussion earlier about mirror neurons, which are also referred to as “embodied simulation”. However, in another study, scientists also found evidence of mirror activity when people observed two inanimate objects touching one another. Here is how they conclude their findings: “…the domain of touch appears not to be limited to the social world. Space around us is full of objects accidentally touching each other, that is, without any animate involvement. One could observe a pine cone falling on the garden bench in the park or drips splashing on the leaves of a plant during a downpour. Models of embodied simulation posit that the same neural structures involved in our own body-related experiences contribute to the conceptualization of what we observe in the world around us” [43].

This is why the activation of architectural events is driven by one’s possession of a body. This is explained by the optic nerve stimulating motor nerves, creating a sympathetic influence on our neural system through the organization of one’s body [44].

### 4.2. Emotional and Behavioral Responses to Environment

The perception of our surroundings is majorly present in our day-to-day life, whether it is through making sense of what we see [14], navigating the spaces around us [30], or triggering a physical reaction [40]. This significant role of perception in our lives makes us wonder, can the spaces surrounding us impact our emotional and behavioral states? Apart from its practical purpose, architecture also has additional cognitive and emotional effects [45]. According to research in the fields of neuroscience and architecture, the emotional responses of individuals can be affected by different interior architectural forms [26]. This effect, which is frequently instinctive and unconscious, emphasizes the close relationship between architectural environments and human emotions. Furthermore, historical references that date back to French architectural theorists of the eighteenth century have highlighted the idea that architecture has the ability to connect to people’s minds and souls [46].

It was shown through multiple studies that architecture and individuals’ mental health are directly correlated [45,47,48]. The acknowledgment that emotions are essential to decision-making, reasoning, and human existence and that empathy is influenced by environmental factors calls for significant changes in both education and the design of our environment [49].

A study showcased significant correlations exist between the experience of a place and the activation of the brain. The study conducted neurological experiments to assess the emotional–perceptual experience of places, revealing significant differences in ratings between pleasant and unpleasant places. Intra-group comparisons indicated that pleasant places were associated with stronger brain activation and a higher number of voxels compared to unpleasant places. The analysis, using voxel-wise and cluster-wise FDR correction methods, demonstrated that pleasant places stimulate the brain and emotion-related regions more than unpleasant places. Overall, the findings suggest that pleasant places have a greater impact on brain activation compared to unpleasant places [47].

Moreover, it was shown that both children and adults who regularly played outside showcased better mental health in comparison with individuals who stayed indoors all the time [48]. This could be a result of exposure to sunlight, which indicates the importance of windows in architectural designs. In fact, the impact of windows with a focus on three factors, sunlight, views, and illumination, was investigated in a study conducted on 100 employees in a manufacturing company in southern Europe [50]. The study showed that the presence of sunlight and nature views increased job satisfaction and decreased stress. Additionally, the study noted a decrease in workers quitting [50]. Accordingly, another study conducted on 333 Dutch office employees noted a reduction in physical and psychological discomfort in workers who had window views [51].

Moreover, a direct correlation between exposure to views, nature, and art and reductions in patients’ stays in hospitals has been proven [52]. One study found that increasing natural light intake and installing a big ceiling-mounted window in a common living area of older persons with cognitive impairment improved day/night orientation and reduced erratic behavior [53]. Studies have also been conducted in places like intensive care units, where it is challenging to establish a natural lighting atmosphere [54]. According to a study, patients in an intensive care unit may experience a return of their melatonin secretion and rhythm when a customized patient-centered light environment is established [55,56]. In general, the creation of an environment that utilizes or simulates natural light can be beneficial to health; however, in circumstances when circadian mismatch cannot be avoided, like shift work, the external environment can be altered to decrease the discrepancy and create a home-like atmosphere [57]. However, natural light is not the only factor in architectural design that impacts individuals’ mental health state; the designs’ colors also play an important role.

A study investigated the impact of color on individuals’ emotions within architectural spaces. Through exposure to virtual environments, participants experienced resting (black), control (white), and chromatic (blue) settings. The results indicated that exposure to the blue environment led to increased respiratory and skin conductance responses compared to the white and black settings. Additionally, there was a rise in alpha frontal midline power and frontal hemispheric lateralization in the blue condition. These results suggested that the color of architectural spaces, particularly blue, can trigger autonomic responses and brain activity linked to emotional processing [58]. Accordingly, it was suggested that color has a profound effect on human physiology and emotions; red boosts blood flow to the muscles, activates the sympathetic nervous system, and raises brain wave activity, all of which quickens breathing, heart rate, and blood pressure. Additionally, the color blue was suggested to have a calming effect, with its ability to stimulate the parasympathetic nervous system [59]. However, it was argued that the color is not important in itself, but its importance rather lies in helping us understand the shapes and forms of any space, suggesting that color is one of the major design elements within architecture [60].

This same paper suggested that architecture is the “third skin” of the human body, with the first skin being their actual skin and the second skin being their clothes [60]. This metaphor is used to highlight how much an individual is affected by the environment they are in. Accordingly, in this sense, the Regio Emilia approach to education considers the built environment to be “the third teacher” [61]. This approach implies that surroundings have their own role in developing and expanding children’s learning. As suggested by the Reggio Emilia approach, teachers should be particularly aware of the various ways that space may be used to “speak” and encourage engagement [62]. Some examples of these techniques include arranging easels near windows or strategically placing small mirrors throughout the classroom. Teachers can use “provocations”, such as a pizza box in the kitchen corner, paper and pencil in the block center, or enticing smells to entice the kids’ senses when they first walk into the classroom, to surprise the kids and start a conversation. Adding actual items for kids to utilize in their play, such as various types of colored and shaped pasta in the house corner, is another tactic [62].

Accordingly, a paper reviewing the architecture and interior designs in Italian kindergartens and their relationship with motor development noted that “the construction of a school is the first pedagogical act” [63]. The paper suggested that the children should be considered as the center of the space that is being built for them. Therefore, a kindergarten or a school should be built for a “child” and not an “adult”, meaning the items within the kindergarten should be of colors that stimulate children’s curiosity, and the windows should be designed for safety but at the same time give children access to a view of the outside environment. The impact of schools’ architecture on individuals is not only limited to children. Two researchers in China conducted a study, through questionnaires, comparing student satisfaction in traditional lecture classrooms and active learning classrooms. The study found that both classrooms required improvements in learning support aspects; for lecture classrooms, space and furniture perception as well as the physical and decorative environment should be taken into consideration, as they boost students’ perceptions in these classrooms, while for active learning classrooms, space perception is the most important factor [64].

However, some architectural designs negatively impact individuals’ mental wellbeing. Multiple designs were demonstrated to increase individuals’ anxiety and blood pressure and increase the risk of infections [52]. Moreover, the human body is known to have an optimal circadian rhythm that has evolved to adjust to its environment. Accordingly, sudden or unprecedented modifications to the environment can potentially be harmful to individuals’ physical and mental wellbeing [65,66]. A good example would be the artificial light that floods contemporary residential and urban settings. The patterns of modern light profiles, which are a blend of artificial light and sunlight, affect the body in a way that means it finds it difficult to distinguish between day and night, and light of different intensities and wavelengths appears and disappears in an unpredictable and erratic manner [67,68].

As a matter of fact, the harmful effect of artificial light on people’s circadian rhythm and overall well-being has been discussed in multiple research papers. Numerous studies demonstrate that exposure to light, particularly at night, alters sleep patterns and decreases melatonin release [69,70]. High levels of artificial light exposure throughout the night might cause problems initiating and maintaining sleep, postpone the sleep phase, and even enhance depressive symptoms due to poor quality of sleep, according to other studies [69,71,72].

Furthermore, aging has been linked to an increase in disorders and diseases such as Parkinson’s disease [73], Alzheimer’s [74], anxiety, and depression [73], among others. Therefore, to tackle these growing issues and make the elderly’s lives easier, architectural designs are being modified according to their physical and emotional needs. Places for the elderly should be protected and filled with greenery [75]. Recent studies showed that plants enhanced the surrounding air quality to some degree, which helped reduce the tiredness brought on by loud noises. The primary reason for the reduction in noise caused by plants is their capacity to absorb, reflect, and deflect sound waves [76]. A closed green belt community generated by the vegetation was shown to be the only way to effectively prevent the transfer of sound energy [77]. Furthermore, afforestation and sensible urban planning could preserve the urban environment and successfully manage noise pollution [78].

## 5. Discussion

This paper aimed to investigate the impact of architecture on our behavior, emotions, and general well-being. It was deemed necessary to first understand how individuals perceive the spaces surrounding them. Thus, the first section of this literature review established a foundational understanding of the relationship between architectural spaces and neurological processes. It was shown that the anterior cingulate cortex (ACC) and parahippocampal place area (PPA) play an important role in interpreting and responding to architectural elements [17,24,25]. This neural engagement with our surroundings reveals a complex visual processing hierarchy, where architecture not only serves as a physical backdrop but also as a dynamic participant in shaping our cognitive and emotional experiences.

Perceiving the spaces around us is not enough; spatial layout and navigability are equally critical. Therefore, the next section of this review showed findings that explain how we navigate the built environment. It was found that incorporating distinctive landmarks within a space significantly aids in wayfinding. These features act as memorable reference points, facilitating the formation of mental maps and enabling easier navigation and orientation within complex-built environments. For example, in a large office building, a uniquely designed staircase or a vibrant mural in a central lobby can serve as a landmark [27]. Moreover, the cognitive map theory’s emphasis on the hippocampus’s role in spatial navigation highlights the need for intuitive architectural designs that support easy navigation, particularly in complex environments like healthcare and educational facilities [30]. This approach is vital in enhancing cognitive function and reducing disorientation, especially for individuals with memory impairments or cognitive challenges [37,38].

These two sections helped build the framework of the interplay between architectural design and human neurological functioning. Then, the last section of this review investigated the emotional and behavioral impact of architectural spaces on individuals, which led to the discussion of important factors that architects should consider when building spaces, advocating for a more empathetic and neuroscience-informed approach to architectural design.

First, the integration of natural elements, particularly sunlight and green spaces, is deemed very important. Studies have shown that natural light and views can significantly improve mental health, reduce stress, and enhance job satisfaction [50,51]. This underscores the necessity for architects to integrate natural lighting and outdoor views, thus fostering a connection with the natural environment.

Additionally, the color schemes employed within architectural designs emerge as powerful tools for influencing emotional and physiological responses. The use of specific colors, like calming blue hues, can create environments conducive to relaxation and mental well-being [58,59]. This insight suggests a thoughtful application of color in spaces intended for stress reduction, such as healthcare facilities or areas designated for relaxation and contemplation.

Moreover, considering architecture as an extension of human identity—the “third skin”—encourages a deeper, more empathetic approach to design [60]. Spaces should resonate with human psychological needs, going beyond aesthetic appeal to create environments that support and enrich human experience. This perspective aligns with the insights gathered from the role of mirror neurons, suggesting that architectural spaces can evoke empathetic and emotional responses, thereby connecting individuals to their environment in a meaningful way [41,43].

However, it is essential to recognize the potential adverse effects of certain design choices. The overuse of artificial lighting, for instance, disrupts natural circadian rhythms, leading to sleep disturbances and increased stress [67,68,69]. Architects must balance artificial and natural light sources to support healthier living environments.

This review also points to the broader impact of architectural design on mental health. Access to outdoor spaces and exposure to nature have been linked to better mental health outcomes [48]. Additionally, the presence of green spaces and plant life not only improves air quality but also offers acoustic benefits, contributing to a more serene and less stressful environment [75,76].

Incorporating these elements into architectural design represents a profound shift towards creating spaces that are not only functional but also therapeutic, enhancing well-being and cognitive function. As architects and urban planners embrace these neuroscience-based insights, they have the opportunity to craft environments that are in harmony with human physiological and psychological needs, thus elevating the overall quality of human life and experience.

## 6. Conclusions

In conclusion, this review underscores the significant role of architectural design in influencing human cognition, emotion, and well-being, integrating insights from neuroarchitecture. At its core, architecture is much more than just a physical construction; it is an extension of who we are, deeply entwined with our psychological identities. The viewpoint of Finnish architect Juhani Pallasmaa [2] is particularly relevant in this context, since it emphasizes how structures serve as pathways for our memories, dreams, and thoughts, as well as serving as barriers between our consciousness and the outside world.

The examination of how individuals see their environment has provided insight into the complex brain systems that underlie visual perception and comprehension. The significant influence of architectural design on human cognitive processes is revealed by our growing understanding of how the brain interprets visual information, which are sensitive to contours, forms, colors, and spatial situations. The intricate interplay between environments and neurological functioning, as evidenced by the roles of the anterior cingulate cortex (ACC) and parahippocampal place area (PPA) [17,24,25], and the impact of environmental factors on mirror neurons [41,43] highlight the profound potential of architecture in shaping human experience.

Moreover, drawing on the understanding of the hippocampal role in spatial orientation provides opportunities for architectural interventions, especially in the context of supporting those experiencing difficulties with spatial disorientation [37,38]. Additionally, the idea of embodied simulation—especially with regard to mirror neurons—offers powerful new insights into the sympathetic reactions elicited by architectural features. This neurological mechanism fosters a deeper connection to architectural places beyond their physical qualities by enabling us to feel and perceive our surroundings [41,43].

Thus, the combination of architectural design with neurological knowledge offers a way to build spaces that support human well-being in addition to meeting practical needs. Neuroscience-based architectural interventions have the potential to improve healthcare facilities, educational settings, urban planning, and other related domains. Techniques like incorporating significant landmarks, improving spatial layouts, and using empathic design approaches can have a good effect on people’s emotional experiences, cognitive capacities, and general quality of life [48].

However, this field, while promising, has its limitations. The complexity of human neurology and the subjective nature of architectural perception means that findings are not universally applicable. Cultural, geographical, and individual variances play significant roles [63,64] and must be considered in practical applications. Moreover, the influence of factors like artificial lighting on circadian rhythms [67,68,69], and the design requirements for specific populations such as children and the elderly [75,76,77,78], underscores the need for a nuanced approach in architecture.

This review, therefore, serves as a foundation for future research in neuroarchitecture. It opens up avenues for more in-depth studies to better understand these interactions and their implications in architectural design. While currently limited by its beginning stage and the variability in human experiences, neuroarchitecture holds the promise of creating more empathetic, health-oriented, and psychologically beneficial environments, ultimately enriching the quality of human life.

## Data Availability

Not applicable.
